# Urotensin II in Invertebrates: From Structure to Function in *Aplysia californica*

**DOI:** 10.1371/journal.pone.0048764

**Published:** 2012-11-08

**Authors:** Elena V. Romanova, Kosei Sasaki, Vera Alexeeva, Ferdinand S. Vilim, Jian Jing, Timothy A. Richmond, Klaudiusz R. Weiss, Jonathan V. Sweedler

**Affiliations:** 1 Beckman Institute for Advanced Science and Technology and the Department of Chemistry, University of Illinois at Urbana-Champaign, Urbana, Illinois, United States of America; 2 Department of Neuroscience, Mount Sinai School of Medicine, New York, New York, United States of America; Medical University Innsbruck, Austria

## Abstract

Neuropeptides are ancient signaling molecules that are involved in many aspects of organism homeostasis and function. Urotensin II (UII), a peptide with a range of hormonal functions, previously has been reported exclusively in vertebrates. Here, we provide the first direct evidence that UII-like peptides are also present in an invertebrate, specifically, the marine mollusk *Aplysia californica*. The presence of UII in the central nervous system (CNS) of *Aplysia* implies a more ancient gene lineage than vertebrates. Using representational difference analysis, we identified an mRNA of a protein precursor that encodes a predicted neuropeptide, we named *Aplysia* urotensin II (apUII), with a sequence and structural similarity to vertebrate UII. With *in-situ* hybridization and immunohistochemistry, we mapped the expression of apUII mRNA and its prohormone in the CNS and localized apUII-like immunoreactivity to buccal sensory neurons and cerebral A-cluster neurons. Mass spectrometry performed on individual isolated neurons, and tandem mass spectrometry on fractionated peptide extracts, allowed us to define the posttranslational processing of the apUII neuropeptide precursor and confirm the highly conserved cyclic nature of the mature neuropeptide apUII. Electrophysiological analysis of the central effects of a synthetic apUII suggests it plays a role in satiety and/or aversive signaling in feeding behaviors. Finding the homologue of vertebrate UII in the numerically small CNS of an invertebrate animal model is important for gaining insights into the molecular mechanisms and pathways mediating the bioactivity of UII in the higher metazoan.

## Introduction

The cyclic peptide urotensin II is considered the most potent mammalian systemic vasoconstrictor and hypertensive agent identified to date [1], [2]. Initially identified from the caudal neurosecretory system of the teleost fish [3], [4], the UII prohormone and its gene were later discovered in phylogenetically higher vertebrates (for review see [5]) such as the green frog [6], mouse and rat [7], [8], monkey [9] and human [7]. The RNA encoding the UII precursor is found in the spinal cord of pig [10], human and frog [11]. In contrast to many of the neuropeptides/hormones in mammals that evolved from ancient versions [12], the mammalian UII gene has not been reported until this study to have homologous peptide systems in invertebrates. This lack is surprising as prohormone databases for insects, worms and crustaceans have rapidly grown, especially due to the recent advent of high throughput genetic sequencing and peptide prediction and identification methods [13], [14], [15], [16], [17], [18], [19], [20], [21]. According to the latest evolutionary analysis, the UII peptide system is thought to have appeared early in vertebrate evolution [22], after the divergence of deuterostomes and protostomes.

Based on mRNA expression and direct mass spectrometric measurement of peptides in individual neurons, here we present the first direct evidence of the existence of UII outside of the Chordata, namely in the protostomian mollusk *Aplysia californica*, thus expanding the history of UII beyond vertebrates.

The high degree of evolutionary conservation of urotensin II across taxa in the animal kingdom suggests that this peptide exerts important physiological actions**.** Indeed, UII is widely expressed in the central nervous system (CNS) of frog [6], in cardiovascular and renal systems, and numerous peripheral organs of mammals [23], [24], [25], [26], where it regulates endocrine, cardiovascular, renal, and immune functions [2], [5], as well as activates a spectrum of behavioral effects [27] including feeding [28]. With its G-protein-coupled receptor [29], [30] located in the heart, lungs, blood vessels, and brain [31], UII is considered a potential target for human pharmacotherapy [1], [24], [26], [32], [33]. Despite its broad occurrence, the existence of specific receptors and the indications of a multiplicity of effects, the functional significance and underlying mechanisms of UII are difficult to clarify in vertebrates due to the inherent complexity of their behavioral and physiological repertoire. The discrepancy between the localization of the mammalian UII mRNA and the mature peptide primarily to motoneurons, [7], [8], [34] and the wide distribution of its known receptor, are frustrating and intriguing at the same time.

Our discovery of the UII prohormone in *Aplysia* creates an opportunity for studying UII actions at the cellular, system and behavioral levels in a well-characterized model organism. Fundamental questions related to the functions of bioactive peptides can be studied conveniently and effectively in *Aplysia* due to its relatively simple nervous system, which allows accessibility to identifiable neurons [35], [36]. Moreover, *Aplysia* is an excellent model for studying the transitions between peptide signaling events occurring at the cellular level, and physiology and behavior at organismal levels [37], [38], [39], [40], [41], [42], [43]. The cellular functions of disease-related molecules have been studied in mollusks as well. For instance, molecules related to Alzheimer’s disease have recently been tested in the gastropod mollusks *Lymnaea stagnalis* [44] and *Aplysia* [45]. Intriguingly, more orthologs and paralogs of genes associated with human disease are reported in *Aplysia* than in other leading model organisms from Arthropoda (*Drosophila melanogaster*) or Nematoda (*Caenorhabditis elegans*), perhaps due to a slower rate of gene evolution [46], [47], [48]. The discovery of a UII peptide in an invertebrate model animal may facilitate elucidation of the functional significance of UII in behavior and numerous pathophysiological conditions, and offer insights into the evolution of highly conserved peptide systems shared with humans.

## Results

### Representational Difference Analysis (RDA) to Identify the apUII Transcript

**Figure 1:**
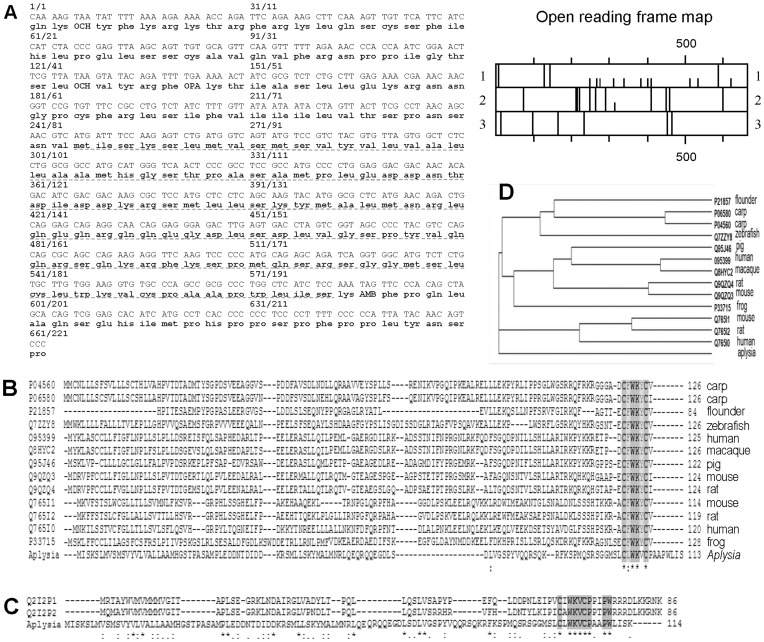
Sequence of the apUII precursor and its comparison to known homologous sequences from vertebrates. (**A**) Consensus cDNA (plain font), translated protein sequence **(bold font).** Right: open reading frame map (full vertical line, stop codon; half line, met, beginning of protein) shows three reading schemes: 1. as read shown at left, 2. shift right by 1 nucleic acid, 3. shift right by 2 nucleic acids. The map shows Scheme 1 is the most appropriate reading scheme. (**B**) A comparison between known UII precursor sequences from different species; for human, mouse and rat, urotensin-2B prohormone sequences (Q76510, Q76511, Q76512, respectively) were included in the alignment in addition to UII sequences For carp, two sequences found in UniProtKB and differing by a few single amino acid substitutions were also included. Protein accession numbers are shown at left. The BLOSUM62 identity scoring is shaded; the lighter shade represents an identity of >50%, darker shade is 100%. Symbols: “*” indicates fully conserved amino acid positions, “:” and “.” indicate strong (>0.5) and weak (≤0.5) positively scoring groups that occur in the Gonnet Pam250 matrix. (**C**) Sequence alignment of apUII and contulakins from the lettered cone snail shows similarity highlighted by the BLOSUM62 method. (**D**) the urotensin II precursor phylogenetic tree calculated by the neighbor-joining method from the multiple sequence alignment.

Because the *Aplysia* genome is currently being sequenced [49], [50], [51], [52], [53], neither the assembled or annotated genome is yet available (http://www.broadinstitute.org/scientific-community/science/projects/mammals-models/vertebrates-invertebrates/aplysia/aplysia-genom). Therefore, neuropeptide prohormones need to be cloned from the CNS. To identify differentially expressed mRNAs that code for neuropeptide prohormones, we performed differential screening using the RDA approach [43]. Because the metacerebral cell (MCC) appears to lack neuropeptides, it makes an excellent “driver” for RDA subtractions. Using two clones from the RDA between the buccal sensory neurons and the MCC, we produced a consensus containing an entire open reading frame of a putative neuropeptide precursor (Fig. 1A) that predicts a neuropeptide (apUII) having sequence similarity to the vertebrate urotensin II. Thus, the prohormone was named apUII due to its shared sequence homology with other known urotensin II peptides in a variety of species (Fig. 1B).

Consistent with the targeting of the peptide precursor to the secretory pathway, the apUII precursor has a hydrophobic signaling sequence at the N-terminus. According to the SignalP signal sequence predictor [54], the signal peptide is most likely cleaved between the A [30] and M [31] residues in the preprohormone.

### Analysis of the apUII Prohormone Structure Confirms Similarity to Vertebrate UII

For the analyses of the prohormone structure, we queried the UniProtKB database with “urotensin II” terms and selected manually annotated (reviewed) entries that were further restricted to the terms “urotensin” in protein name and “2” in protein family. The 19 UniProtKB identifiers were restricted by the range of sequence lengths to exclude incomplete prohormones and sorted by organism. The remaining 12 identifiers were aligned with ClustalW [55]; for rat, mouse and human, two prohormones were included in the alignment, urotensin-2 and -2B. The alignment showed conserved amino acids in the cyclic portion of the apUII peptide where four positions were identical (including the position of Cys residues), and eight positions were similar across the compared species (Fig. 1B). A phylogenetic tree was generated on the basis of multiple sequence alignments by the neighbor-joining [56] (Fig. 1D).

Similar to other known mature UII isoforms [1], apUII contains a conserved cyclic hexapeptide core sequence (CFWKYC). This cyclic domain is the minimal sequence that retains full biological activity [28] and is structurally similar to that in the functionally important central region of somatostatin-14 (FWKT) [57]. While this peptide sequence is well conserved, the rest of the prohormone sequences differ considerably from vertebrate analogs. BLAST analysis with the entire apUII precursor showed no significant alignments in the non-redundant database, suggesting that apUII is a novel precursor. BLAST analysis of the apUII peptide sequence revealed a 50–75% similarity to the contulakin-Lt1 and conlulakin-Lt2 peptides (Fig. 1C) from the lettered cone snail *Conus litteratus* [58].

### Mapping the Expression of apUII in the CNS with *In-situ* Hybridization

Identification of the apUII precursor sequence allowed us to use *in situ* hybridization to determine the distribution of the neurons that contain apUII mRNA in the CNS of *Aplysia*. *In situ* hybridization was performed using the antisense cRNA (generated from the whole sequence as illustrated in Fig. 1A) made with T7 or SP6 RNA polymerase (n = 3). We also performed vector control experiments where only vectors were included, and the antisense apAUII cRNA sequence was omitted. These controls resulted in no staining.

**Figure 2:**
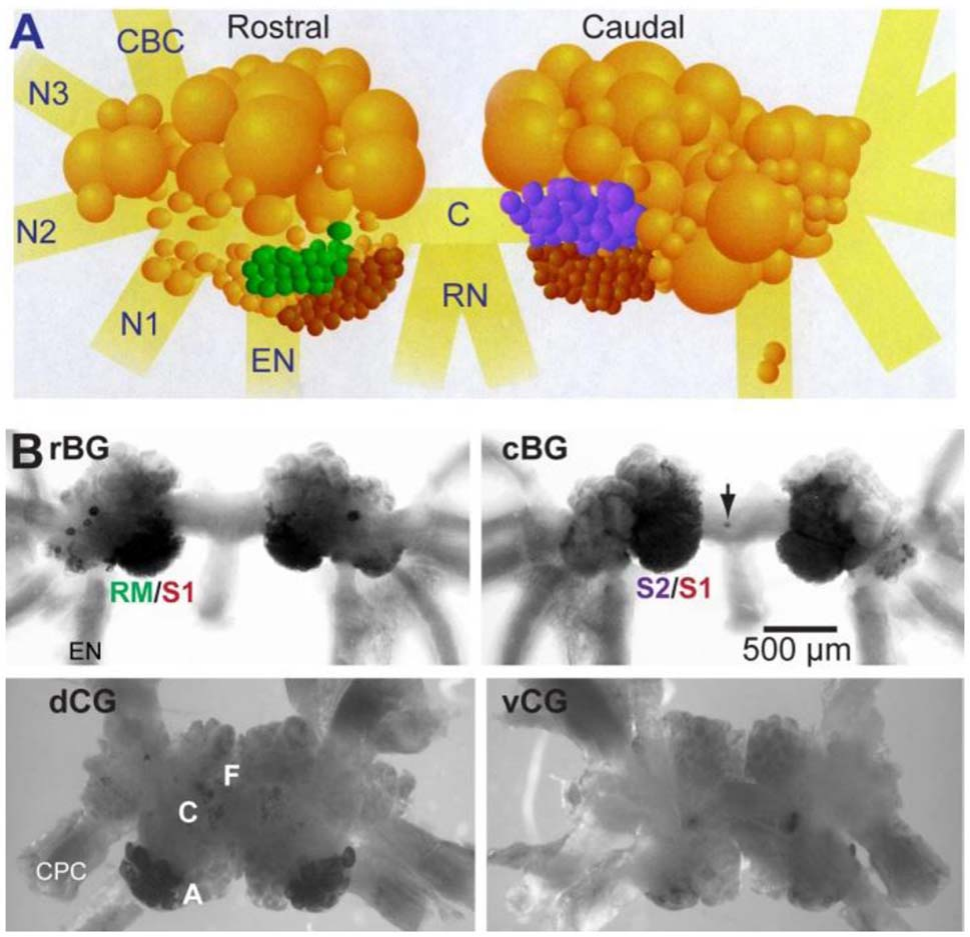
Distribution of apUII positive neurons in the buccal and cerebral ganglia. (**A**) Diagram of the buccal ganglion neurons involved in generating feeding behavior, highlighting the sensory neuron cluster. The larger motoneurons are present as a cluster on the dorsal surface of the ganglion (the larger orange cells above), while the smaller sensory neurons are present in a cluster on the dorsal-medial aspect of the ganglion. The population of buccal sensory neurons is heterogeneous. They are grouped into at least three clusters with distinct sensory properties and transmitter contents. The radula mechanoafferent neurons (green) innervate the subradular tissue and contain SCP and enterin. The S1 sensory neuron cluster (purple) is present only on the caudal surface and is distinguished by a slightly larger size than the S2 sensory neuron cluster (brown). The S1 cluster contains primarily FMRFamide, while S2 contains primarily FRFamide. Here, we show that apUII is expressed in both the S1 and S2 clusters. Note that the commissure is twisted, so that on the left, the rostral surface of the buccal ganglion is shown, while the right side shows the caudal surface. Nerve abbreviations, Com: buccal commissure; CBC: cerebral-buccal connective; EN, esophageal nerve; N, buccal nerve; RN: radular nerve. (**B**) *In-situ* hybridization staining of the buccal and cerebral ganglion shows the distribution of apUII mRNA expressing neuron somata. The buccal sensory neurons express the mRNA for apUII. Abbreviations, rBG: rostral side of the buccal ganglion; cBG: caudal side of the buccal ganglion; dCG: dorsal side of the cerebral ganglion; vCG: ventral side of the cerebral ganglion. S1, S2, A-, F- and C-neuronal clusters are labeled.

In the present study, we focused on two central ganglia, the buccal and the cerebral, which are involved in the generation of feeding behaviors. Multiple apUII mRNA-positive neuronal somata were observed in both the buccal and cerebral ganglia (Fig. 2). In the buccal ganglion (Fig. 2B, rBG, cBG), strong staining was found in the sensory S1 and S2 clusters [59], [60], seen on both the caudal and rostral surfaces (Fig. 2A), a small group of neurons (arrowhead in Fig. 2B, rBG) in the vicinity of the cerebral-buccal connective, and a number of distributed neurons asymmetrically located on the rostral side of both hemiganglia. A small individual neuron (arrow) was stained in the caudal buccal commissure near the radula nerve. In the cerebral ganglion (Fig. 2B, dCG, vCG), intense staining was present in both hemiganglia in the area corresponding to the A-clusters and was mostly seen on the dorsal surface, while a weak staining was localized to the dorsal middle section of the ganglion in the area of the F- and C-clusters.

### Mass Spectrometric Analysis of Prohormone Processing and Posttranslational Modifications (PTMs)

**Figure 3:**
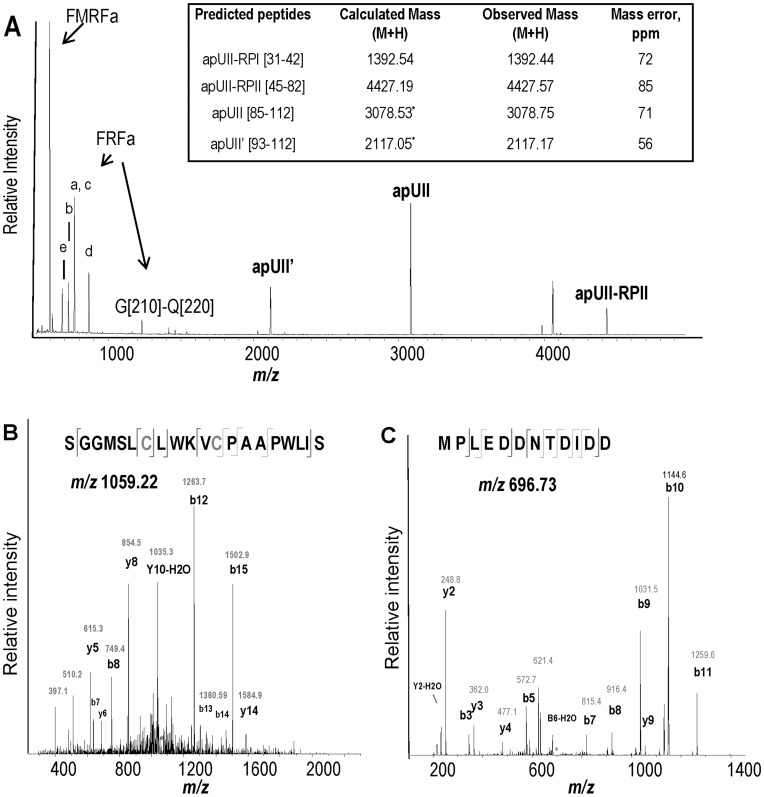
Mass spectrometric analysis of apUII prohormone processing and structure verification. (**A**) Representative MALDI MS spectrum of individual cultured buccal sensory neurons expressing FMRFa, FRFa and apUII prohormones. apUII-derived peptides (in bold), FMRFa, FRFa peptides (a–e) and an apUII linker peptide are labeled. The inset table specifies the accuracy of the apUII peptides measurement; (M+H) is the monoisotopic mass of the protonated molecular ions, (*) denotes the theoretical mass corrected for the difference from the disulfide bond. (**B**) Fragmentation spectrum of apUII peptide S [93]-S [112] detected in a +2 charge state with the two red Cys residues highlighted (lower case c). (**C**) Fragmentation spectrum of apUII peptide S [45]-Q [82] detected in a +2 charge state.

Knowing the translated apUII precursor sequence (Fig. 1B), we employed matrix-assisted laser desorption/ionization (MALDI) mass spectrometry (MS) to directly demonstrate the presence of apUII and determine the posttranslational processing of its precursor at the level of specific neurons. To aid the confirmation of new neuropeptides using MS, we first entered the prohormone sequence into the NeuroPred processing prediction tool (http://neuroproteomics.scs.illinois.edu/cgi-bin/neuropred.py) [61]. This revealed a 99%, 98% and 75% cleavage probability at basic amino acids K [43]–R [44], K [83]–R [84], and K [102], respectively, in the apUII precursor sequences. The probability of cleavages at the other basic sites was <50%. NeuroPred also generated a list of peptides potentially resulting from proteolytic processing. MALDI-time-of-flight (TOF) MS was used to analyze the neuropeptides from the sensory neurons of the buccal ganglion (Fig. 3A). Cleavage of the signal peptide between the A [30] and M [31] residues was confirmed by detection of peptide M [31]–D [42]. Both dibasic consensus cleavage sites found on the apUII prohormone and one monobasic site with the highest predicted probability were confirmed by the MALDI MS detection of corresponding peptides M [31]–D [42] (*m/z* 1392.4), S [45]–Q [82] (*m/z* 4430.0), and F [85]–S [112] (*m/z* 3080.7) (see Fig. 1B), in the buccal sensory neurons (BSNs) (Fig. 3A). Peptide F [85]–S [112], which contains the conserved sequence and referred to as apUII, was detected with a mass 2 Da smaller than its theoretical mass, which suggests a PTM in the form of a disulfide bond between the C [99] and C [104] residues. The apUII peptide is further processed at the monobasic site R [92] (predicted cleavage probability: 46%). The resulting truncated peptide that retains the conserved sequence, S [93]–S [112] (*m/z* 2118.6), named apUII’, was detected with a delta mass of 2 Da, which further supports the attribution of the 2 Da mass discrepancy on a larger peptide to the formation of disulfide bonding. The other two peptides were named as apUII-RPI (apUII related peptide I) for M [31]–D [42] and apUII-RPII for S [45]–Q [82]. Potential products of apUII precursor cleavage at other monobasic or unconventional sites were not detected. The data are summarized in the inset table shown in Fig. 3A. Additional masses observed in these samples were assigned to known prohormone-related products–FMRFa- and FRFa-related peptides [60], [62], [63], small cardioactive peptides (SCPs) [59], [64] and sensorin [65], all shown to be expressed in BSNs. Peptide profiles indicative of expressed prohormones differed between cultured neurons, confirming the previously reported non-uniform biochemical composition of the BSN clusters [60]. Our single-cell MALDI MS analysis of freshly isolated neurons and peptide extracts from cerebral F- and C-clusters did not reveal the presence of apUII peptides, although their presence in those locations was suggested previously on the basis of immunostaining with antibodies raised against a non-*Aplysia* UII [66].

To obtain an independent verification of the structure of the apUII peptide, we performed tandem MS peptide sequencing on fractionated extracts from cerebral A-clusters and BSNs. Fragmentation of the [93]SGGMSLCLWKVCPAAPWLIS [112] putative peptide that was observed as a doubly charged ion at *m/z* 1059 verified the sequence corresponding to apUII (Fig. 3B). Both a long form (apUII [85–112]) and a short form (apUII’ [93–112]) of the apUII neuropeptide were detected, as well as peptide apUII-RPII (Fig. 3C). One feature of UII is its cyclic structure. The presence of the intramolecular disulfide bond on the apUII to create the cyclic peptide structure was verified via tandem MS. Complementary ions series containing the C [99]–C [104] loop were observed, therefore confirming a conserved cyclic peptide structure (Fig. 3B).

### Localization of apUII Immunoreactivity in the CNS

**Figure 4:**
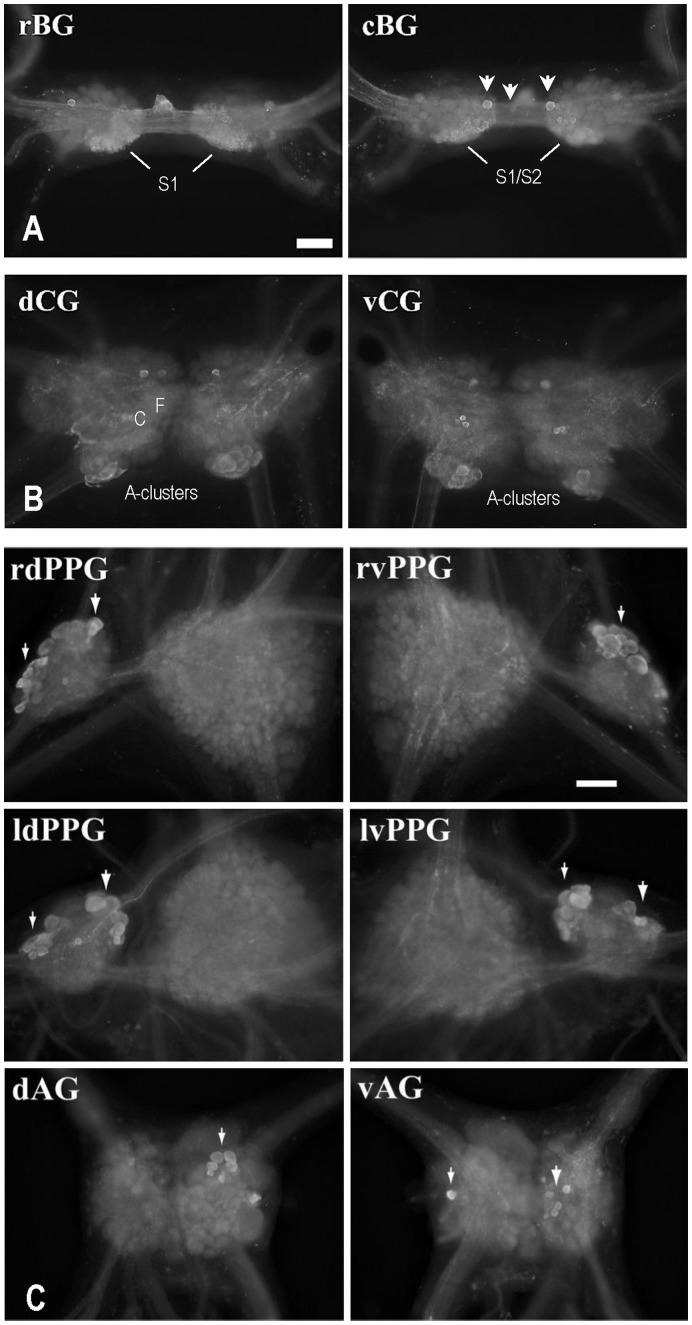
Immunostaining of apUII-positive neurons and fibers in the Aplysia CNS. (**A**) The distribution of immunostained neurons in the buccal ganglion is similar to the *in-situ* hybridization (ISH) staining distribution shown in Figure 2B. Abbreviations, rBG: rostral surface of the buccal ganglion; cBG: caudal surface of the buccal ganglion. Arrowheads point to individual stained neurons. **(B**) The distribution of immunostained neurons in the cerebral ganglion is similar to the ISH staining distribution shown in Figure 2B. Abbreviations, dCG: dorsal surface of the cerebral ganglion; vCG: ventral surface of the cerebral ganglion; CPC cerebral-pedal connective; A, C, F, neuronal clusters. (**C**) The distribution of immunostained neurons in the pedal, pleural and abdominal ganglia. Abbreviations, rdPPG: right dorsal pleural-pedal ganglia; ldPPG: left dorsal pleural-pedal ganglia (for these two images, the pleural ganglia are on the left); rvPPG: right ventral pleural-pedal ganglia; lvPPG: left ventral pleural-pedal ganglia (for these two images, the pleural ganglia are on the right); dAG: dorsal abdominal ganglion; vAG: ventral abdominal ganglion. Arrowheads point to stained neurons. Calibration bar for all panels : 200 µm.

Immunostaining using an antiserum generated against the 14-aa N-terminal peptide from apUII with sequence [85]FKSPMQSRSGGMSL [98] was performed to localize the neurons and processes that recognize anti-apUII antibody (n = 4). To determine the specificity of the antibody, we performed adsorption control where the peptide was first incubated with the antibody, which resulted in no staining. Moreover, the staining patterns of the antibody (Fig. 4A) matched well with the staining from the *in situ* hybridization experiments (Fig. 2B), which further supported the specificity of the antibody.

As expected, the S neurons of the BSNs (S1 and S1) were immunoreactive for the apUII antibody (Fig. 4A, rBG, cBG). In addition, two symmetrical, larger neurons on the caudal side in the vicinity of the S-clusters (arrowhead in Fig. 4A, cBG) were brightly immunoreactive, as well as a lone neuron in the buccal commissure (arrowhead). Likewise, on the rostral side, two symmetrical, larger neurons at the origin of the buccal peripheral nerves showed apUII immunoreactivity, along with S-cluster immunoreactive fibers noted in the radula nerve, cerebral-buccal connectives and buccal peripheral nerves. It appears that radula mechanoafferent neurons [59], known to innervate subradular tissue, were also slightly immunoreactive to apUII.

In the cerebral ganglion (Fig. 4B, dCG, vCG), the apUII immunoreactive somata were observed on both the ventral and dorsal sides. The immunoreactivity was localized to A-clusters and three sparse neurons in the vicinity of the G-clusters. There was scarce immunostaining in the region of the F- and C-clusters. Diffuse fibrous and punctuate staining appeared on the right dorsal surface of the cerebral ganglion. A few smaller, medium-sized neurons were immunoreactive in the middle section and two symmetrical neurons in the vicinity of the G-cluster border on the ventral side of the cerebral ganglion were prominent. Taken together, apUII, identified using RDA and MS, is present in the *Aplysia* CNS, providing a basis for the functional studies described in the next section.

In addition to the buccal and cerebral ganglia, we also performed apUII immunostaining on two other central ganglia: pleural-pedal and abdominal ganglia, which do not play a major role in feeding. In the pleural-pedal ganglia (Fig. 4C), most apUII immunopositive neurons (arrows) were localized in the pleural ganglia, with virtually no staining in the pedal ganglia. In the abdominal ganglia (Fig. 4C), several apUII immunopositive neurons (arrows) were observed on both the dorsal and ventral surfaces.

### Physiological Activity of apUII in the Feeding Circuit

Expression of the mammalian UII receptor in the rat brain has suggested an involvement of UII in feeding behavior [31]. In physiological experiments, we used apUII that contains a highly conserved cyclic C-terminus formed by covalent binding between 2 Cys residues (see Fig. 3). We have not tested apUII-RPI and apUII-RPII, which are not conserved with vertebrate UII, but are potentially bioactive.

**Figure 5:**
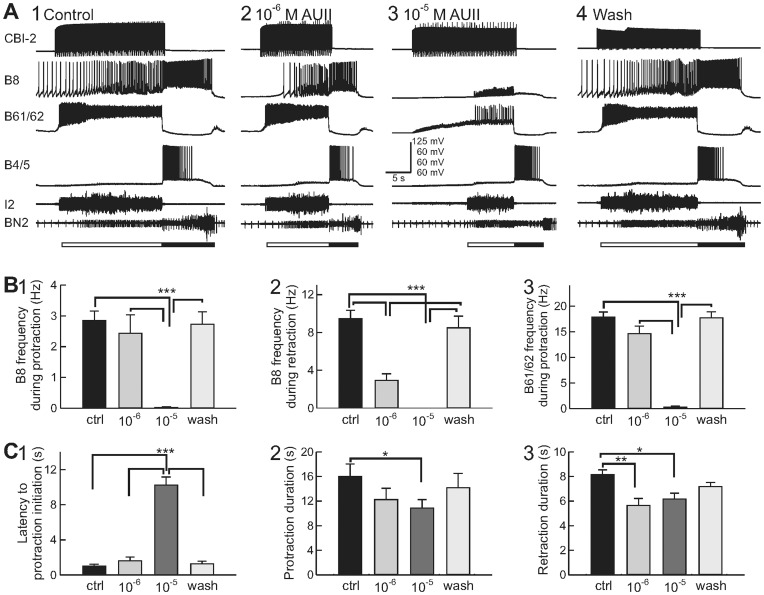
apUII actions on motor programs elicited by a command-like interneuron CBI-2. (**A**) Representative examples. CBI-2 was stimulated throughout the protraction phase (open bar) to elicit single cycle motor programs; the inter-trial interval is 1 min. In control condition (**A1**), B8 is predominantly active during the retraction phase (filled bar), while B61 is only active during protraction. Perfusion of 10^−6^ M (**A2**) and 10^−5^ M (**A3**) apUII’ reduced the firing frequency of both B8 and B61, but had a lesser effect on the firing frequency of B4/5. Protraction is monitored via activity of I2, retraction via activity of BN2. (**B**) Group data showing the suppressive effects of 10^−6^ M and 10^−5^ M apUII’ on B8 firing frequency during protraction (**B1**), or during retraction (**B2**), and B61/62 firing frequency during protraction (**B3**). (**C**) Group data showing the lengthening of the latency to protraction initiation, measured as the time that elapsed from the onset of CBI-2 stimulation to the onset of I2 nerve activity (**C1**). apUII’ also reduced protraction duration (**C2**) and retraction duration (**C3**). *, p<0.05; **, p<0.01; ***, p<0.001; error bars represent SEM.

**Figure 6:**
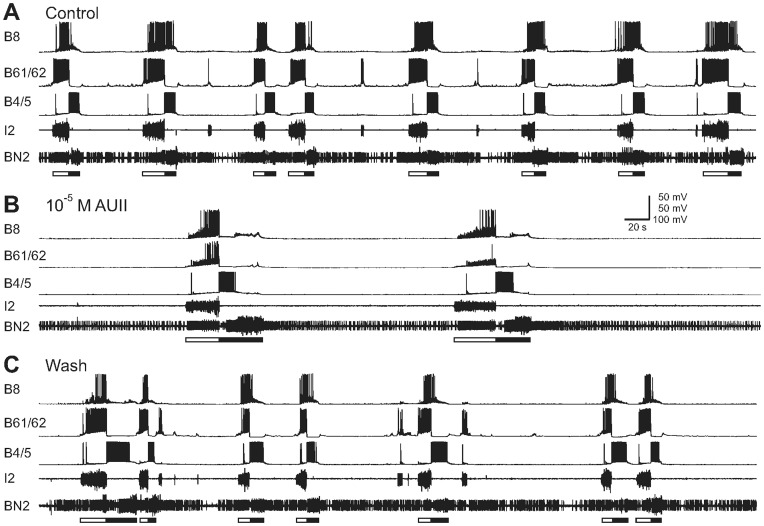
apUII inhibits the generation of spontaneously occurring programs. (**A**) In control condition, motor programs occurred spontaneously. (**B**) Perfusion of 10^−5^ M apUII reduced the frequency of motor programs. (**C**) Upon washout of the peptide, motor programs occurred more frequently. Each cycle of motor programs consists of a protraction (open bar) and retraction (filled bar) sequence.

In *Aplysia*, the feeding network is known to be extensively modulated by neuropeptides [38], [39], [40], [42], [43], [60], [67], [68], [69], [70], [71]. Here, we examined the possible modulatory actions of apUII in the *Aplysia* feeding network. The feeding motor programs are generated by a central pattern generator (CPG) located in the buccal ganglion [72], [73]. All feeding motor programs consist of a protraction-retraction sequence. The radula closure motoneuron B8 can be active during protraction or retraction, depending on the type of motor programs. In ingestive programs, B8 is predominantly active during retraction; while in egestive programs, B8 is predominantly active during protraction. In isolated CNS preparations (Figs. 5A and 6), protraction was monitored via activity in the I2 nerve and in protraction motoneuron B61/62. Retraction was monitored by large unit activity in buccal nerve 2 (BN2) and the depolarization of neurons B4/5 that follows protraction. Radula closure was monitored by activity in B8.

In the first series of experiments, we examined whether apUII had any effect on the motor programs elicited by stimulation of a command-like interneuron, CBI-2, located in the cerebral ganglion (Fig. 5A). CBI-2 was stimulated at 10 Hz for the duration of protraction to elicit single cycle motor programs. In the control condition, B8 was predominantly active during retraction, thus the program was ingestive. Perfusion of the synthetic peptide (apUII’[93–112] internal disulfide bonded) reduced the activity of several motoneurons, including B8 (n = 9, protraction: F_(3,24)_ = 27.32, p<0.0001; retraction: F_(3,24)_ = 38.1, p<0.0001) and B61/62 (n = 4, F_(3,9)_ = 141.1, p<0.0001), in a concentration-dependent manner (Fig. 5B). Inhibition of CPG activity was also reflected as an increase in the latency to protraction initiation (Fig. 5C1, F_(3,15)_ = 115.6, p<0.0001, n = 6). Perfusion of apUII also resulted in a small but statistically significant shortening of protraction (Fig. 5C2, F_(3,15)_ = 4.77; p<0.05, n = 6) and retraction (Fig. 5C3, F_(3,15)_ = 7.51; p<0.01, n = 6) duration.

**Figure 7:**
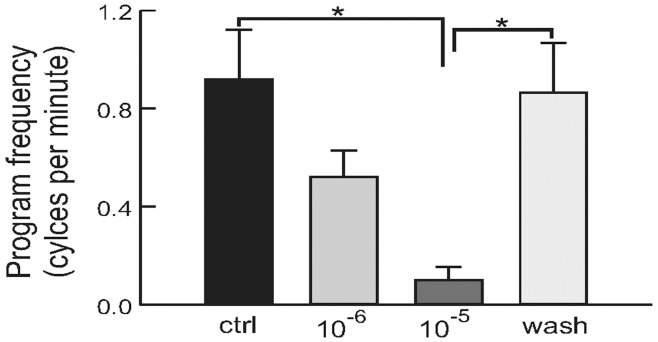
Summary data showing the effects of 10^−6^ M and 10^−5^ M apUII on the frequency of spontaneously-occurring motor programs. The frequency of motor programs is the number of cycles per minute measured over a period of 10 minutes or more in each experimental condition. There is a dose-dependent reduction in the frequency of spontaneously-occurring motor programs. *, p<0.05; error bars represent SEM.

In addition, we took advantage of the fact that in some preparations, the CPG can also be spontaneously active in the absence of any stimulation. We found that perfusion of apUII reduced the frequency of spontaneously occurring programs (Figs. 6 and 7, F_(3,6)_ = 7.63; p<0.05, n = 3). Taken together, these results provide evidence that apUII suppresses the generation of feeding motor programs.

## Discussion

The earlier the origin of a specific peptide system, the greater the probability that paralog lineages exist because of subsequent genome expansion events. Thus, determining whether a specific peptide is present in invertebrates should help to explain the existence of peptides having a wide-range of actions. Multiple peptide genes with known biological functions in mammals are also found in invertebrates, such as those encoding FMRFamide-like peptides and neuropeptide Y (NPY); both FMRFamide and NPY are also found in protostomian flatworms and arthropods [74], and their cDNA and mRNA have been reported in mollusks [75], [76]. The RFamide peptide family can be traced as far as back as the cnidaria (hydra) [77] and insulin, and opioid peptide precursors have been reported for unicellular organisms [78].

According to an evolutionary analysis of urotensin peptide systems, UII is thought to have appeared in chordates after the divergence of deuterostomes and protostomes [22], [79]. Among lower vertebrates, UII has been reported in jawless fish such as lamprey [80], which lack a caudal neurosecretory system. Isolation of UII peptides from whole brain extracts of the sea lamprey, *Petromyzon marinus*, and the river lamprey, *Lampetra fluviatilis*, which are structurally identical to UII from the dogfish [81] and skate [82], suggests that the primary structure of UII was well-conserved phylogenetically among the ancient vertebrates where it may have functioned as a neurotransmitter/neuromodulator in the CNS, rather than as a neurohormone of the caudal neurosecretory system [80]. Here, we present evidence that the UII prohormone is expressed and can fulfill a signaling function in the protostomian mollusk *A. californica.* This expands the history of UII beyond vertebrates. To demonstrate the close similarity to the vertebrate peptide, we structurally characterized mature processed peptides and confirmed the existence of the conserved cyclic structure in both the long and the short versions of the peptide that we therefore named *Aplysia* urotensin II, or apUII. Our preliminary findings were presented at the annual SFN meeting [83]. To the best of our knowledge, other reported analyses of posttranslational UII prohormone processing were done in flounder [84] and human [85].

The first suggestion of a urotensin-like substance present in *Aplysia* dates back to 1985 when Yui et al. [86] reported immunoreactivity to vertebrate urotensin I (UI) antisera in nerve fibers of the muscle layer of the gut, but not in the endocrine paraneurons. The same study suggested the presence of urotensin-like immunocytochemistry in two other protostomians, silkworm and cricket [86]. Later, the presence of a fish urotensin-like substance was suggested by urotensin-like immunoreactivity in the CNS of *Aplysia* [87], [88]. However, these early observations have not been followed up with biochemical peptide characterizations or gene discovery efforts. Likewise, *Aplysia* was one of the first models used to study the modulatory actions of UII. Sawada and Ichinose [89] reported that when commercial synthetic vertebrate urotensin I and II peptides were applied at “physiological” concentrations, they produced a decrease of neuronal excitability by potentiating the GABA(A) receptor-mediated Cl^-^ current in *Aplysia* neurons in a manner suggesting a modulatory, rather than a direct, peptide action. Notably, a small change in the structure of a peptide may lead to activation of different receptors. UI, a fish analog of mammalian corticotropin-releasing factor, has a distinct structure. UI and UII are thought to originate from different ancient ancestors [9], [57], [79], [90]. Thus, the effects of vertebrate UI and UII on *Aplysia* neurons should be interpreted cautiously.

Vertebrate UII and the UII-related peptides (URPs) are thought to originate from the same ancestral gene as the somatostatin and cortistatin genes [4], [57], [91], [92]. Interestingly, **s**omatostatin-like material was found in flowering plants and in *Escherichia coli* grown in defined medium, and it reacted only to C-terminal immunoassays [93], [94]. The latest data indicate that the UII/URP and somatostatin genes arose through a segmental duplication of two ancestral genes physically linked to each other, which in turn arose through a tandem duplication of a single ancestral gene. Some authors suggest that URPs and somatostatin-related peptides may belong to the same superfamily [91]. Our reconstruction of the phylogenetic tree based on multiple UII sequence alignments (Fig. 1D) suggests that the apUII prohormones originate from an ancestral lineage shared with mammalian UII prohormones and thus, may belong to the same superfamily. However, additional data are needed to fully understand apUII gene evolution. Furthermore, apUII has a similarity with contulakin peptides, an atypical, non-cysteine-rich conopeptide group that contains only one cysteine bond [95]. Our findings suggest that the first duplication of an ancestral UII gene may have occurred at a much earlier time in metazoan evolution than the emergence of the teleost fish [57].

As discussed in detail in recent reviews [5], [24], due to the potent vasoconstrictor and hypertensive properties of UII in mammals, the focus of many functional studies of UII has been on its endocrine/paracrine actions. However, the broad distribution of UII receptor in the CNS–most recently reviewed by Hunt et al. [96] and do Rego et al. [28]–suggests that this peptide may act as a neuromodulator in sleep regulation, anxiety and behavior. Due to the complex organization of the mammalian brain, it is difficult to study the mechanisms of peptide action. Furthermore, suggestions about the central actions of UII have often been based on the expression patterns of UII receptors and/or pharmacological actions of UII [5]). Interestingly, the expression of a UII receptor in brain regions controlling appetite, food consumption and energy homeostasis [31], as well as studies with intracerebroventricular UII administration [27], suggest a role for UII in modulating feeding behavior. In our study, we take advantage of the numerically small nervous system of *Aplysia* to characterize the physiologic actions of UII and demonstrate directly that apUII produces powerful cellular and network actions within the animal’s feeding circuit.

Elaborate physiological mechanisms control feeding behavior, including those dependent on the interplay between neuropeptidergic pathways. It has been shown that intracerebroventricular injection of UII into the lateral ventricle of mice induces a dose-dependent increase in both food and water intake [97]. The range of active doses of UII is similar to the range that induces anxiogenic- and depressant-like behaviors [28], [97]. Neuroanatomical studies on the distribution of UII receptors in the brain provide additional evidence for the involvement of UII and/or URP in the control of appetite and food consumption [27], [31].

Here, we validate the modulatory role of apUII in the *Aplysia* feeding network. We show that apUII exerts inhibitory actions on the feeding CPG. These actions are similar to those exerted by FRFamides, another family of neuropeptides expressed in the BSNs [60] (Fig. 2A). The inhibitory actions of the neuropeptides contained in the BSNs suggest that apUII-expressing neurons may have a function in aversive behaviors that include inhibition of feeding responses. Interestingly, the inhibitory actions of these peptides may also contribute to the implementation of a satiety state. Our previous work [40] suggests that the early stage of the satiation process entails generation of more egestive programs that reduce food intake. This stage is followed by an eventual termination of feeding. Indeed, other sensory neuron peptides, SCP [98] and FMRFamide [60], promote generation of egestive programs, while apUII and FRFamide may contribute to the eventual suppression of feeding. Thus, some peptides of the BSNs (Fig. 2A) may act in concert to produce satiety. In summary, discovery of these important peptides in an invertebrate model animal may be expected to facilitate studies that aim to uncover regulatory mechanisms of highly conserved signaling peptidergic systems that lower animals share with humans.

## Materials and Methods

### Animals

For molecular and electrophysiological experiments, *A. californica* weighing 10–20 g were obtained from the University of Miami *Aplysia* Research Facility (Miami, FL) and 100–350 g animals were obtained from Marinus (Long Beach, CA) and Pacific Biomarine (Venice, CA, USA). For analytical experiments, the 80–150 g animals were purchased from Charles Hollahan (Santa Barbara, CA, USA). The animals were kept at 14°C in 150-gallon tanks with circulating artificial seawater (ASW) prepared from Instant Ocean (Aquarium Systems, Mentor, OH, USA). For electrophysiological experiments, animals were used within three weeks of arrival. Prior to dissection, animals were anesthetized by an injection of 30–50% (w/v) of isotonic magnesium chloride into the visceral cavity. Isolated CNS were immediately processed for sample preparation. Cell cluster classification was performed according to previously reported methods [99], [100].

### Representational Difference Analysis

Recently we adapted the RDA system [101], [102] to identify novel peptides from single identified neurons in the *Aplysia* CNS, as described in detail previously [43]. In brief, the entire procedure can be divided into three steps: (1) Isolation of two types of identified cells. The first is the cell of interest, or “tester”, whose peptides are to be identified; the second cell, or “driver”, is used to subtract sequences that are shared with the tester. The rationale behind this RDA approach is that the tester shall contain one or more peptides that are not present in the driver. Multiple cells from each cell type are collected in a solution of ice cold 50% propylene glycol, 1.2 M NaCl in diethylpyrocarbonate-treated H_2_O, and stored in −80°C. (2) Amplification of the cDNA from the RNA [103] of the tester and the driver. (3) RDA [101], [102] with the amplified cDNA of the tester and the driver. The cDNAs of the driver and tester were digested with DpnII, and the driver cDNA was ligated to R-Bam adaptors, while the tester cDNA was ligated to N-Bam adaptors. The driver DNA was amplified with biotinylated R-Bam primer, and then hybridized with unamplified tester cDNA. Here, we modified the RDA by including a physical subtraction based on biotinylated driver primers, streptavidin incubation and phenol/chloroform extraction [104]. This procedure removed the driver cDNA bound to either the driver cDNA or the tester cDNA because the driver cDNA contained biotinylated adaptor. We then performed the final amplification with tester N-Bam primer to further enrich the sequences that were unique to the tester.

### *In-situ* Hybridization

*In-situ* hybridization was performed as described previously [103]. Ganglia were digested with 1% protease type IX (Sigma-Aldrich, St. Louis, MO, USA) in 10 mL of artificial sea water (ASW: 460 mM NaCl, 10 mM KCl, 55 mM MgCl_2_, 11 mM CaCl_2_, and 10 mM HEPES, pH 7.6) for 3 h at room temperature (with rocking) to facilitate the removal of the sheath. After digestion, the ganglia were washed with ASW and fixed overnight at 4°C with 4% paraformaldehyde (Electron Microscopy Sciences, Fort Washington, PA, USA) in phosphate-buffered saline (PBS). The ganglia were then washed, desheathed, dehydrated in an ascending ethanol series, and then rehydrated. Following rehydration, the ganglia were prehybridized and then hybridized overnight at 50°C in a buffer solution (50% formamide, 5 mM ethylenediaminetetraacetic acid (EDTA), 5× saline-sodium citrate, 1× Denhardt’s solution, 0.1% Tween 20, and 0.5 mg/mL yeast tRNA) containing 2 µg/mL digoxigenin-labeled cRNA probes made from apUII cDNA templates. Following wash-out of the probes, the ganglia were then incubated overnight at 4°C with a 1∶200 dilution of alkaline phosphatase-conjugated anti-digoxigenin antibody (Roche Molecular Biochemicals, Indianapolis, IN, USA) in PBS containing 0.1% Tween, 0.2% bovine serum albumin (BSA) and 1% normal goat serum [PBT]. After washes with PBT to remove unbound antibody, the ganglia were washed with detection buffer (100 mM NaCl, 50 mM MgCl_2_, 0.1% Tween 20, 1 mM levamisol, and 100 mM Tris-HCl, pH 9.5) and developed with 4.5 µL of nitroblue tetrazolium and 3.5 µL of 5-bromo-4-chloro-3-indolyl phosphate (Roche Molecular Biochemicals) in 1 mL of detection buffer. The staining reaction was monitored visually and stopped by washing with PBT when the level of staining was adequate. The stained ganglia were photographed using a Nikon microscope (Morrell Instruments, Melville, NY, USA) with epi-illumination against a white background. Photographs were taken with a Nikon CoolPix 990 digital camera (Nikon, USA), imported into Photoshop (Adobe Systems Incorporated, San Jose, CA, USA) and compiled into figures.

### Cell Isolation and MALDI MS

Using the apUII *in situ* hybridization map as guidance, we located the BSN clusters (see Fig. 2A) and cerebral A-clusters in semi-intact ganglia by their relative position, morphology and size. Following treatment with 1% protease IX (Sigma-Aldrich) in ASW supplemented with antibiotics and a 3-h wash in ASW, the ganglionic sheath was removed and individual BSNs were manually dissociated from isolated clusters. Protease treatments done to a whole isolated CNS in order to digest the outer connective tissue around it do not penetrate into the cell body and therefore, do not affect the dense core vesicles where endogenous neuropeptides are packaged. Using a plastic micropipette, isolated neurons were immediately transferred into a culture dish containing a poly-D-lysine-treated indium tin oxide coated glass slide (Sigma-Aldrich) in ASW, and incubated at +14°C overnight. Then glycerol was added to the dish at a final concentration of 30%, the entire liquid was removed by aspiration, the slide with cultured neurons was air dried for a few minutes, and the location of individual neurons noted with a permanent marker on the bottom of the slide. A MALDI matrix solution (2,5–dihydroxybenzoic acid, 30 mg/mL in 50% acetonitrile (ACN), 0.01% trifluoroacetic acid) was applied over the entire slide using an air brush (Paasche Airbrush, Chicago, IL, USA). Peptide standards were spotted on the corners of the slide.

Cell samples were analyzed by an ultrafleXtreme mass spectrometer equipped with smartbeam and TOF/TOF technologies (Bruker Daltonics, Billerica, MA, USA). Positive ion mass spectra were generated in reflectron mode at a 1 kHz repetition rate. Each representative mass spectrum was a sum of 500–1000 laser shots. We used NeuroPred [61] and the SignalP signal sequence predictor [54] to calculate the predicted peptides and aid in interpreting the mass spectra.

### Purification and Mass Spectrometry of A- and S-Cluster Homogenate

Following dissection, protease treatment and desheathing of the CNS, cerebral A- and buccal S-cluster neurons were manually isolated from four CNS and homogenized in 40 µL of acidified acetone (40∶6∶1, acetone:water:concentrated HCl, by volume) with a plastic homogenizer. The homogenate was centrifuged at 10,000 × g, and the supernatant removed and dried in a Speed Vac (ThermoSavant, Holbrook, NY, USA). The concentrated sample was then diluted to 10 µL with 0.1% formic acid (FA) and loaded onto an Acclaim® PepMap100 Nano-Trap column (200 µm × 2 cm, 5 µm particle size, 300 Å pore size) at a 20 µL/min flow rate of loading solvent (98% H_2_O, 2% ACN, 0.05% FA) and desalted for 5 min using a Dionex UltiMate 3000 Rapid Separation LC system with a capillary flow selector. The sample was then separated on an Acclaim® PepMap100 C18 column (300 µm × 150 mm, 5 µm, 100 Å) at a uniform flow rate of 4 µL/min with an eluting gradient of 15–50% solvent B (B: 90% ACN, 10% H_2_O, 0.04% FA (v/v); A: 0.05% FA (v/v)) over 50 min. The eluent was directly connected to the electrospray ionization (ESI) probe on an HCT Ultra PTM Discovery System (Bruker Daltonics). The ESI-MS measurements were performed in the data dependent acquisition mode with dynamic exclusion at full scan from *m/z* 300 to 2000, and a collision-induced dissociation scan at 35% energy. Fragmentation spectra were deconvoluted and exported as Mascot generic files for identification via database search using Peaks Studio 5.3 (Bioinformatics Solutions Inc., Waterloo, ON, Canada). For the database search we used an in-house *Aplysia* database of known published and putative predicted prohormones. The spectra matching the apUII-derived peptides were manually verified for the presence of complimentary ion series confirming the predicted sequence.

### Antibodies and Immunostaining

The apUII N-terminal peptide sequence FKSPMQSRSGGMSL was used to generate the antibodies in rats as described previously [38], [39]. Briefly, the antigen was prepared by coupling synthetic peptide (SynPep, Dublin, CA, USA) to BSA (catalog #A0281; Sigma-Aldrich) using 1-ethyl-3-(dimethylaminopropyl)carbodiimide (catalog #E7750; Sigma-Aldrich). The coupled antigen was purified and used to inoculate the rats. Immunocytochemistry was performed as described previously [42]. Tissues were fixed in freshly prepared fixative (4% paraformaldehyde, 0.2% picric acid, 25% sucrose, and 0.1 M NaH_2_PO_4_, pH 7.6). Tissue was permeabilized and blocked by overnight incubation in blocking buffer (10% normal donkey serum, 2% Triton X-100, 1% BSA, 154 mM NaCl, 50 mM EDTA, 0.01% thimerosal, and 10 mM Na_2_HPO_4_, pH 7.4). The primary antibody was diluted 1∶250 in the blocking buffer and incubated with the tissue for 4–7 d. The tissue was then washed twice per day for 2–3 d with washing buffer (2% Triton X-100, 1% BSA, 154 mM NaCl, 50 mM EDTA, 0.01% thimerosal, and 10 mM Na_2_HPO_4_, pH 7.4). After the washes, the tissue was incubated with a 1∶500 dilution of secondary antibody (lissamine rhodamine donkey anti-rat; Jackson ImmunoResearch, West Grove, PA, USA) for 2–3 d. The tissue was then washed twice with the washing buffer for 1 d and four times with storage buffer (1% BSA, 154 mM NaCl, 50 mM EDTA, 0.01% thimerosal, and 10 mM Na_2_HPO_4_, pH 7.4) for 1 d.

### Analysis of Electrophysiological Activity in the Feeding Circuit

Intracellular and extracellular recordings of physiological activity from the CNS preparations (including the cerebral and buccal ganglia) were performed as previously described [105]. The ganglia were desheathed, transferred to a recording chamber containing ∼1.5 mL of ASW, continuously perfused at 0.3 mL/min, and maintained at 14–17°C. Peptides were dissolved in ASW immediately before each application and the ASW with the peptide was perfused into the recording chamber. Intracellular recordings were obtained using 5–10 MΩ sharp microelectrodes filled with 2 M K acetate and 0.3 M KCl.

Electrophysiological recordings were digitized online with Axoscope (Molecular Devices, Inc., Sunnyvale, CA, USA) and plotted with CorelDraw (Corel Inc., Mountain View, CA, USA). Bar graphs were plotted using SigmaPlot (Systat Software Inc., San Jose, CA, USA). Data are expressed as mean ± SEM. Statistical tests (repeated-measures of one-way ANOVA) were performed using Prism (GraphPad Software, La Jolla, CA). When data showed significant effects in ANOVA, further individual comparisons were performed with Bonferroni’s correction.
